# Superstorm Sandy exposure *in utero* is associated with neurobehavioral phenotypes and brain structure alterations in childhood: A machine learning approach

**DOI:** 10.3389/fnins.2023.1113927

**Published:** 2023-02-02

**Authors:** Gozde M. Demirci, Donato DeIngeniis, Wai Man Wong, A. Duke Shereen, Yoko Nomura, Chia-Ling Tsai

**Affiliations:** ^1^The Graduate Center, City University of New York, New York, NY, United States; ^2^Queens College, City University of New York, New York, NY, United States

**Keywords:** machine learning, explainable AI, prenatal maternal stress, Superstorm Sandy, brain volume, child behavior

## Abstract

**Introduction:**

Prenatal maternal stress (PNMS), including exposure to natural disasters, has been shown to serve as a risk factor for future child psychopathology and suboptimal brain development, particularly among brain regions shown to be sensitive to stress and trauma exposure. However, statistical approaches deployed in most studies are usually constrained by a limited number of variables for the sake of statistical power. Explainable machine learning, on the other hand, enables the study of high data dimension and offers novel insights into the prominent subset of behavioral phenotypes and brain regions most susceptible to PNMS. In the present study, we aimed to identify the most important child neurobehavioral and brain features associated with *in utero* exposure to Superstorm Sandy (SS).

**Methods:**

By leveraging an explainable machine learning technique, the Shapley additive explanations method, we tested the marginal feature effect on SS exposures and examined the individual variable effects on disaster exposure.

**Results:**

Results show that certain brain regions are especially sensitive to *in utero* exposure to SS. Specifically, *in utero* SS exposure was associated with larger gray matter volume (GMV) in the right caudate, right hippocampus, and left amygdala and smaller GMV in the right parahippocampal gyrus. Additionally, higher aggression scores at age 5 distinctly correlated with SS exposure.

**Discussion:**

These findings suggest *in utero* SS exposure may be associated with greater aggression and suboptimal developmental alterations among various limbic and basal ganglia brain regions.

## 1. Introduction

The prevalence of prenatal maternal stress (PNMS) has increased alarmingly; a recent large-scale study reported approximately 30% of pregnant women reported one or more types of stressors [Loomans et al., 2013; reviewed in Van den Bergh et al. (2020)]. Further, climate change events, in the form of hurricanes, tropical storms, wildfires, flooding, and droughts, are increasing in frequency and becoming more extreme in nature. These weather events cause serious disruptions in people’s lives and pose important threats to the mental health of individuals, especially among vulnerable populations such as pregnant women (Clemens et al., 2020; Zakrison et al., 2020). Considering the prevalence rate of PNMS, it is imperative to investigate its impact on early neurobehavioral development in their offspring (Monk et al., 2019).

An accumulating number of studies have established that various forms of PNMS serve as a risk factor for future child psychopathology (Monk et al., 2019; Lautarescu et al., 2020; Van den Bergh et al., 2020; Nomura et al., 2022). This included *in utero* maternal stress due to exposure to weather-related disasters, such as the Quebec Ice Storm, which led to greater externalizing and internalizing clinical behaviors (King et al., 2012) and Superstorm Sandy (SS), which led to greater clinical and adaptive behaviors (Nomura et al., 2022). Other studies have investigated the biophysiological consequences of suboptimal neurobehavioral development using magnetic resonance imaging (MRI) (Buss et al., 2010; El Marroun et al., 2016; Lebel et al., 2016; Davis et al., 2017; Wen et al., 2017; Mareckova et al., 2022, reviewed in Lautarescu et al., 2020). MRI serves as an unparalleled technology to pinpoint the structural and functional brain changes in various brain regions of the limbic system, such as the amygdala and hippocampus, and frontal lobe, such as the prefrontal cortex, among offspring exposed to PNMS and how those changes underlie the consequent neurobehavioral, emotional, and cognitive changes observed. Machine learning (ML), a rapidly developing technique in data science, is defined as a robust data-driven approach to automatically detect underlying patterns in high-dimensional data with high accuracy (Bi et al., 2019). Several studies have adopted ML algorithms and identified an enlarged amygdala as an important risk factor for early childhood anxiety (Qin et al., 2014), while other studies have discovered a wide range of biopsychosocial causal features using a predictive classification model (Saxe et al., 2017). However, research on the application of ML onto both neurobehavioral and brain volumetric data has been very limited in pediatric populations (Oskar and Stingone, 2020).

To date, there are a small number of ML studies pertaining to the association between prenatal stress or trauma exposure and altered neurobehavioral development and brain volumetric changes of offspring. One study demonstrated detection and classification of prenatal alcohol exposure (Rodriguez et al., 2021) and another identified the most affected regions of the brain (volumes) in children exposed to alcohol prenatally (Little and Beaulieu, 2020). However, a gold-standard of randomization is not possible among human populations, nor had any other work investigated disaster related PNMS in a quasi-experiment model in a human population. Quasi-experimental designs make it possible to pseudo-randomize prenatal stress independent of confounding personal attributes, such as genetic makeups and maternal psychological disorders status (Lafortune et al., 2021). SS, which hit a wide region in New York City (NYC), randomly “assigned” stressful conditions to pregnant women and their offspring, constructing an objective measure of stress independent of the mothers’ genetic background, psychopathology, and socioeconomic status. Thus, the Stress in Pregnancy (SIP) study, with its uniform exposure to a stressor, SS, enabled us to address the inherent bias that traditionally plagues studies assessing the impact of stress in a human population. As such, by leveraging the technique of explainable ML, this study attempts to identify important child neurobehavioral, and brain volumetric features associated with natural disaster-related PNMS; explainable ML makes it possible to uncover patterns of high-dimensional neurobehavioral and brain data of children exposed to such disasters.

## 2. Methodology

The study capitalized on a longitudinal study that followed mother/child dyads from *in utero* to age 11, who were exposed, or not exposed, to Superstorm Sandy (SS) that hit metropolitan New York in 2012. An ML classifier was built to identify neurobehavioral and brain volumetric features that set a child with *in utero* exposure to SS apart from those without. The binary *in utero* exposure to a natural disaster was formulated as the target of the classification problem. ML can facilitate identification of features that dominate the classification process leading to high prediction accuracy. These features can be understood as the phenotypes most affected by natural disaster-related PNMS and studying them helps understand which behaviors and brain volumetric data in the selected brain regions are most affected as a result of SS exposure *in utero*. To this end, we trained a classification ML algorithm for features associated with SS exposure on a given participant and applied explainable artificial intelligence (AI) to identify features that can more accurately contribute to predicting structural changes in brain volume from the exposure.

### 2.1. Study population

Established in 2009, participants were drawn from the SIP study, a longitudinal study that follows mother/child dyads (from *in utero* to age 11) who were exposed, or not exposed, to SS (Finik and Nomura, 2017). Mothers of the current study cohort were originally recruited from antenatal OB/GYN clinics in New York City in efforts to understand how *in utero* exposure to adversity may alter fetal growth and development (Finik and Nomura, 2017). In 2013, a subsample of the SIP cohort, who were exposed or not exposed to SS *in utero* (*N* = 350), was analyzed to explore the trajectories of neurobehavioral development in offspring prospectively. Reflecting metropolitan New York, the children of the SIP study represent an urban population encompassing a diverse range of ethnic/racial backgrounds and socioeconomic strata; the majority of the cohort includes underrepresented races (Black, Hispanic, and/or Asian) and financial minorities (low socioeconomic status (SES), living below the poverty line) (Finik and Nomura, 2017). As a pilot study, a subsample of 30 were contacted for enrollment in MRI procedures. The MRI sample consisted of thirty school-aged children (*n* = 30) with a mean (SD) age of 8.50 (1.98). From the total sample, 21 (7 males and 14 females) were unexposed and 9 (1 male and 8 females) were exposed to SS *in utero*. All participants provided written consent and the protocol was approved by the Institutional Review Boards at the City University of New York (CUNY). Inclusion criteria for the original SIP study included being pregnant at the time of recruitment and planning to deliver the baby. Exclusion criteria for original participation included HIV infection, maternal psychosis, maternal age <15 years, life-threatening maternal medical complications, and congenital or chromosomal abnormalities in the fetus. Further details of the study can be found elsewhere (Finik and Nomura, 2017). Additionally, in this pilot, exclusion criteria for MRI participation included metal implants, devices, and/or objects in the body. Information on the handedness of the children was not collected. All participants provided written consent; the protocol was approved by the Institutional Review Boards at the City University of New York.

### 2.2. Measures

#### 2.2.1. Neurobehavioral functioning

Child neurobehaviors were measured at age five (mean = 4.51, SD = 0.77) using the 2nd edition of the Behavioral Assessment for Children Parent-version (BASC-2P, Reynolds and Kamphaus, 2004). The BASC-2P produces eight clinical and four adaptive profiles. Based on the age and sex of the child, scores were standardized with a mean of 50 and a SD of 10 (Reynolds and Kamphaus, 2004). The eight clinical dimensions were Hyperactivity, Aggression, Anxiety, Depression, Somatization, Atypicality, Withdrawal, and Attention Problems. The four adaptive dimensions were Adaptability, Social Skills, Activities of Daily Living, and Functional Communication. Internal consistency in all 12 sub-dimensions were acceptable (α > 0.80) (Reynolds, 2010). The mean clinical and adaptive neurobehavioral scores by SS exposure status are shown in Table 1.

**TABLE 1:** Mean clinical and adaptive neurobehavioral scores by SS exposure.

		Superstorm Sandy status^a^	
Behavior	Total sample (*n* = 30)	Not exposed (*n* = 21)	Exposed (*n* = 9)
Clinical scales	Mean (SD)	Mean (SD)	Mean (SD)
Hyperactivity	47 (9.33)	46 (8.15)	50 (11.50)
Aggression	45 (8.83)	43 (5.92)	50 (12.44)
Anxiety	52 (8.95)	51 (8.87)	53 (9.59)
Depression	46 (12.20)	45 (10.41)	50 (15.77)
Somatization	47 (8.45)	47 (9.55)	47 (5.55)
Atypicality	51 (10.57)	50 (10.56)	52 (11.04)
Withdrawn behavior	49 (8.58)	49 (7.70)	50 (10.85)
Attention problems	51 (10.85)	49 (10.89)	53 (10.84)
**Adaptive skills**			
Adaptability	45 (10.92)	45 (10.93)	46 (11.47)
Social skills	54 (10.85)	54 (12.56)	54 (5.69)
Activity of daily living	54 (9.17)	56 (6.82)	52 (13.27)
Functional communication	51 (8.95)	50 (9.11)	53 (8.69)

SD, standard deviation. Values represent *T*-scores (Mean = 50; SD = 10); Greater scores on clinical values and lower scores on adaptive values indicate greater impairment.

^a^Prenatal exposure to Superstorm Sandy.

#### 2.2.2. MRI neuroimaging

Magnetic resonance imaging images were acquired using a Siemens 3 Tesla Prisma MRI Scanner. 3D high-resolution T1-weighted images were collected using a magnetization with the following parameters: inversion time (TI)/repetition time (TR)/echo time (TE) = 1,070/2,500/2.9 msec, flip angle = 8.0 degrees, field of view = 256 mm × 256 mm, matrix = 256 × 256, and slice thickness = 1 mm without gap. The number of slices is 176. Real-time motion detection and correction was implemented using Volumetric Navigators (vNav) (Tisdall et al., 2012).

The FreeSurfer pipeline was used to generate cortical and subcortical volumetric measures (Dale et al., 1999). The skull was stripped from the T1 images and the interface between the white and gray matter was estimate and further refined to obtain the thickness of gray matter. Cortical surfaces were inflated and Talairach transformation was performed. The cortex was parcellated into different anatomical regions using Destrieux atlas. The brain regional volumes were normalized by the total intracranial volume. The mean gray matter volumes of the four brain regions most strongly associated with SS exposure are shown in Table 2.

**TABLE 2:** Mean gray matter volume of brain regions associated with Superstorm Sandy exposure.

			Superstorm Sandy status^a^			
Brain region	Total (*n* = 30)		Not exposed (*n* = 21)		Exposed (*n* = 9)	
	Left hemi. mean (SD)	Right hemi. mean (SD)	Left hemi. mean (SD)	Right hemi. mean (SD)	Left hemi. mean (SD)	Right hemi. mean (SD)
Caudate vol.	0.33 (0.04)	0.34 (0.04)	0.32 (0.04)	0.33 (0.04)	0.35 (0.05)	0.37 (0.04)
Hippocampus vol.	0.33 (0.03)	0.34 (0.03)	0.32 (0.02)	0.33 (0.03)	0.34 (0.04)	0.36 (0.04)
Amygdala vol.	0.13 (0.01)	0.14 (0.01)	0.12 (0.01)	0.13 (0.01)	0.13 (0.01)	0.14 (0.01)
PHG vol.	0.21 (0.03)	0.20 (0.02)	0.21 (0.03)	0.20 (0.01)	0.21 (0.03)	0.19 (0.03)

Brain Vol. = % volume normalized by intracranial volume.

PHG, parahippocampal gyrus; Hemi, hemisphere.

^a^Prenatal exposure to Superstorm Sandy.

### 2.3. Machine learning

The primary goal of the study is to evaluate whether certain features in behavioral and brain indices contribute to detecting *in utero* exposure to SS. The classification model of ML was implemented to identify which category (SS or non-SS exposure) the participant belonged to. Three common machine learning algorithms Random Forest (RF), XGBoost, and AdaBoost were evaluated for their ability to predict disaster exposure and identify the independent features that significantly contribute to the model’s decision. The evaluation can be found in Appendix. RF classifier was selected for this study due to its superior performance in prediction accuracy. RF classifier consists of multiple decision trees trained with different sub-portions of the trained dataset to ensure generalizability. The predictions from each subset are then voted for final classification result.

Our data suffers from the curse of dimensionality, where the sample size (*n* = 30) is relatively small compared to the number of variables (45 predictors). In ML implementations, dimensionality increases with each variable in the data. High dimensionality on a very small sample size leads to model overfitting. To avoid overfitting data, we applied a feature selection approach, Recursive Feature Elimination (RFE), to reduce dimensionality (Sartori et al., 2018). RFE starts with all features and eliminates the least important ones until the classifier reaches the best subset for the desired number of features. Reducing the number of features also helps the model to be more generalizable when used in independent data (Watts et al., 2021).

Our sample also suffers from class imbalance with a ratio of 30/70 with a disaster exposed minority group. With imbalance data, classifier models are prone to predict the majority class. We avoided such a problem by creating augmented data to balance both classes using the Synthetic Minority Oversampling Technique (SMOTE) (Chawla et al., 2002). SMOTE randomly perturbs a sample of the minority class based on its k-nearest neighbors to augment a new synthetic sample. Such operation is repeated until the data is balanced in target value classes.

**FIGURE 1:**
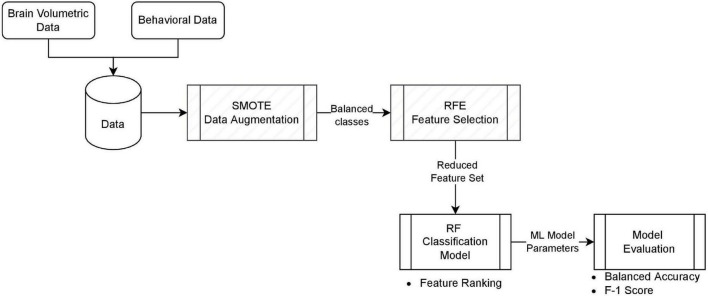
The classification model. Each block represents the implementation component, and the bullet of items is the outcome of that block. Hatch blocks are optional, depending on the model setting.

The process of our classification model is summarized in Figure 1. Brain volumetric data and behavioral data were combined to form our dataset with a total of 45 features. The RF classifier was first trained with all features on the dataset for feature ranking. RFE was applied to extract the most important subset of features based on the ranking and RF was trained again with only these important predictors. We studied four settings with different combinations of data augmentation and feature selection for the best prediction outcome. The four settings are: Model 1.a without SMOTE using all features, Model 1.b with SMOTE using all features, Model 2.a without SMOTE using best feature subset, and Model 2.b with SMOTE using best feature subset. For the models with data oversampling, SMOTE was only applied to the training set.

To facilitate interpretation of our classification model output, an explainable AI approach, Shapley additive explanations (SHAP), was used. Explainability in machine learning refers to the ability to understand and interpret the output of a complicated model. It is an important consideration when developing a machine learning model to ensure transparency to experts in the application domain. Specifically, Shapley Additive exPlanations (SHAP) is a method for explaining the output of machine learning models. It is a game-theoretic approach that assigns each feature of a model a “contribution” value, which represents the magnitude of the effect that the feature has on the model’s output. In other words, SHAP explains the correlation between a given feature and the prediction. In this way, we may call the result explainable ML. SHAP calculates the contribution of any given feature on the target value. For each individual feature x, a calculated SHAP value gives the direction of likelihood of the target value. We can also derive dependence plots from SHAP to show the effect of a single predictor on the prediction made by the model. The visualization of partial dependence plot helps to detect the point where the target value changes from 0 to 1 (or vice versa).

### 2.4. Model validation

Given a small training dataset, the almost unbiased estimate of the true error can be obtained using Leave-One-Out Cross Validation (LOOCV). With a data size of n, LOOCV leaves 1 sample for testing and uses the other n-1 samples for training in each run, and the average accuracy of n runs is reported as the final evaluation score. It is important to note that, for Models 1.a and 2.a, SMOTE was applied only in the training set–there is no synthetic sample in our test data for the purpose of evaluation. We measured the model performance for each setting with balanced accuracy and F1 score results. Balanced accuracy was calculated as the average of sensitivity and specificity, where sensitivity = TP ÷ (TP + FN) and specificity = TN ÷ (TN + FP). The F1 score was calculated with the harmonic mean of precision and recall, where precision = TP ÷ (TP + FP) and recall = TP ÷ (TP + FN). TP = true positives, TN = true negatives, FP = false positives, and FN = false negatives. Here our TP is correctly predicted SS exposure and TN correctly predicted non-SS exposure. F1 score is preferred over balanced accuracy because the true negative correctly predicting non-Sandy exposure is not considered in the computation. Every case where SS exposure was not predicted correctly is penalized. For this reason, our RFE also applied F1-score to decide the best subset of features for feature selection.

## 3. Results

**FIGURE 2:**
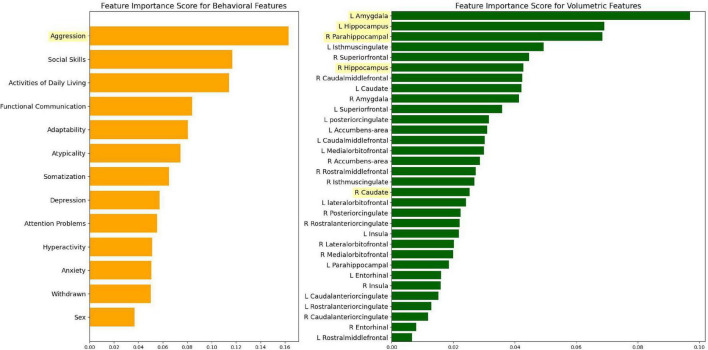
Ranked feature importance according to different variable categories.

Figure 2 shows the plots of ranked feature importance scores for different categories of features: neurobehavioral and brain volumetric data. The most relevant neurobehavioral feature that distinguished SS exposure was aggression. Moreover, the left amygdala, left hippocampus, and the right parahippocampal gyrus (PHG) were the three brain regions that distinguished SS exposure.

### 3.1. Performance analysis

The best subset of six features chosen by RFE include aggression, the right caudate, right and left hippocampus, left amygdala, and right PHG. It is clear from the RFE selected features that brain volumetric data contributed more toward the prediction of the ML model.

**FIGURE 3:**
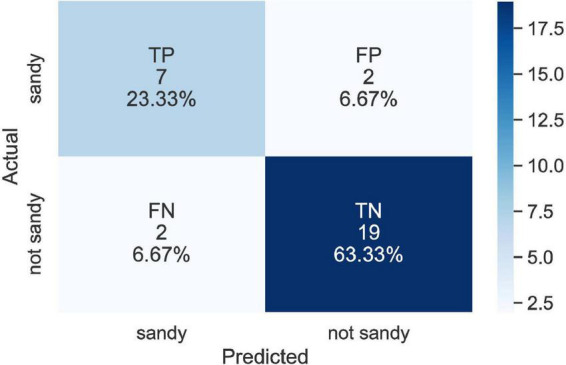
Confusion matrix for the best result (model 2.a).

Table 3 shows each model’s evaluation score. The best result was reached with an F1 score of 0.78 (see model 2.a), developed with the six features chosen by RFE. In Figure 3, the confusion matrix for model 2.a reveals that our model is fairly accurate at detecting both SS exposed and unexposed groups. Of note, when issues related to overfitting is controlled for, SMOTE is no longer effective.

**TABLE 3:** Machine learning approach results.

	Feature selection	Smote	Balanced accuracy	F1-score
1.a	All features (45)	No	0.7333	0.3333
1.b	All features (45)	Yes	0.7333	0.4286
2.a	RFE selection (6)	No	0.8667	0.7778
2.b	RFE selection (6)	Yes	0.8333	0.7058

### 3.2. Feature analysis

**FIGURE 4:**
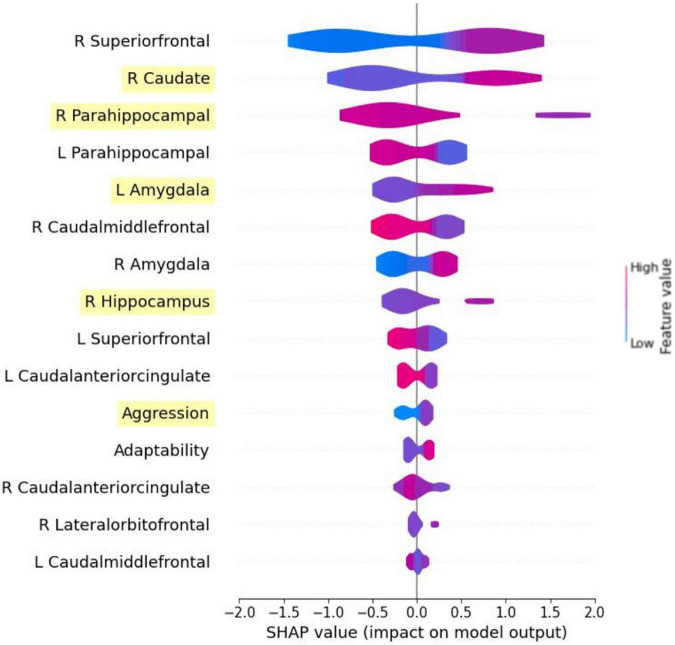
Summary plot in SHAP generated from the model using all features. Only the top 15 features are shown. Features highlighted are those selected from RFE method. To interpret the SHAP plot, the warmer color of the feature bars represents the higher feature values, and the cold color represents the lower feature values. When the SHAP value is higher and ¿0 the model is more likely to predict 1 (Sandy exposure), and when the value is lower and <0 the model is more likely to predict 0 (non-Sandy exposure).

Figure 4 shows SHAP values of the top 15 features from the model trained with all features to demonstrate the model explainability. With the exception of aggression and adaptability, the features that were identified as important were brain regions. The top features included the right superior frontal gyrus, right caudate, right PHG, left PHG, left amygdala, right caudal middle frontal gyrus, right amygdala, right hippocampus, left superior frontal gyrus, left caudal anterior cingulate, aggression, adaptability, right caudal anterior cingulate, right lateral orbitofrontal gyrus, and the left caudal middle frontal gyrus. The features are ordered by their importance score. Five of the six features selected by RFE were also included and ranked in the same order. They are the caudate, right PHG, left amygdala, right hippocampus, and aggression. For the selected features from the RFE method, the SHAP plot revealed that higher aggression, a larger right caudate, smaller right PHG, larger right hippocampus, and a larger left amygdala distinctly showed higher likelihood of SS exposure. In contrast, we see that a larger left and right PHG have a higher chance of predicting non-SS exposure. Furthermore, lower aggression distinctly shows non-SS exposure.

**FIGURE 5:**
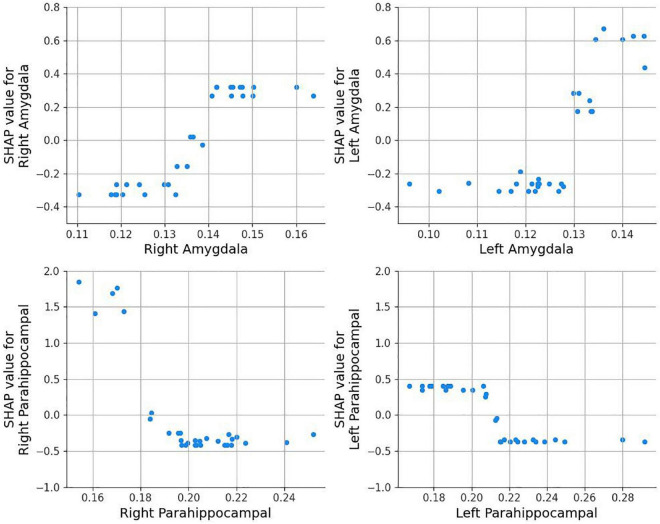
Amygdala and parahippocampal dependence plots. A higher SHAP value indicates that the model is more likely to predict 1 (Sandy exposure).

**FIGURE 6:**
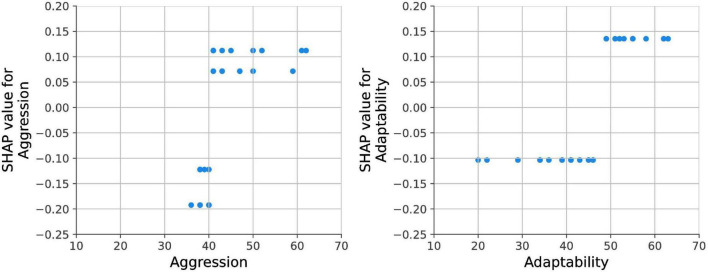
Aggression and adaptability dependence plots.

In Figure 5, the dependence plots for the left and right hemispheres of both the amygdala and PHG (in volume) were given. For the right and left amygdala (top left and right), as the volume increases, the chance for SS exposure also increases. In contrast, for the right and left PHG (Figure 5, bottom left and right), the chance for SS exposure increases as the brain volume decreases. Aggression and adaptability dependence plots are shown in Figure 6. In both features, SS exposure is associated with a higher T score. Notably, for aggression scores, a more explicit difference is found between the SS exposed and unexposed groups.

## 4. Discussion

To the best of our knowledge, the current study is the first to show that the implementation of a machine learning model was predictive in identifying children exposed *in utero* to natural disasters based on various neurobehavioral phenotypes and brain regions, shown to be sensitive to stress and trauma exposure (Lautarescu et al., 2020; National Scientific Council on the Developing Child, 2020; Van den Bergh et al., 2020).

Our results showed distinctly higher aggression and adaptability scores among our SS exposed group, which, consistent with the work of King et al. (2012) and Nomura et al. (2022), suggests *in utero* maternal stress, from exposure to a weather-related disaster, is strongly associated with child neurobehavioral phenotypes. Our results demonstrate the stress a child experiences *in utero* increases the risk for future psychopathology but may also impact a child’s potential to acquire and express certain adaptive skills in their postnatal environment (Nomura et al., 2022).

In agreement with the findings from numerous earlier studies, our results suggest *in utero* stress exposure may be associated with a larger amygdala volume (Buss et al., 2012; Wen et al., 2017; Acosta et al., 2019; Jones et al., 2019) and a larger hippocampal volume (Cao-Lei et al., 2021), although less supported. It has been further suggested that prenatal stress exposure appears to accelerate the development of these regions, particularly the amygdala, as a means of allowing more rapid detection of incoming danger and threat (Lautarescu et al., 2020). Our results are consistent with the evolutional model of stress that suggests the exaggeration of automatic fear detection programmed prenatally to better prepare for the likely at stake environment postnatally (Glover, 2011).

Our results offer further, more novel, insights as *in utero* SS exposure was found to be linked with both a larger caudate and a smaller PHG. Enlargements in the caudate have been suggested to underlie the pathophysiology of various neurobehavioral and emotional disorders, characterized by repetitive and ritualistic tendencies, such as obsessive-compulsive disorder (OCD) and autism (Ring and Serra-Mestres, 2002). A reduced PHG was found to be associated with elevated prenatal maternal anxiety (Buss et al., 2010; Acosta et al., 2019). The caudate and PHG are two brain regions shown to influence emotion regulation and the formation of emotional memories (Bhatt et al., 2012; Zhu et al., 2019; Driscoll et al., 2022). Our findings on a larger caudate and a smaller PHG may extend the current literature and support the association between prenatal natural disaster-related maternal stress and altered child volumetric brain development, which may have long-term implications on child neurodevelopment and emotion regulation (Talge et al., 2007; Wu et al., 2022).

Of note, no notable sex differences were observed between the assessed clinical and adaptive neurobehaviors nor between the GMV of the SS sensitive brain regions. In efforts to explore the association between the GMV of the SS sensitive brain regions and aggression scores, a *post hoc* analysis was conducted. Upon stratification of the sample by the SS exposure status, higher aggression scores were negatively associated with left amygdala GMV [*r*(19) = −0.45, *p* = 0.04] among the SS unexposed and marginally associated with left amygdala GMV among the SS exposed [*r*(7) = −0.60, *p* = 0.09]. No other notable associations between aggression and the GMV of SS sensitive brain regions were detected. We anticipated seeing a strong correlation between amygdala GMV and aggression scores as previous work among a sample of children, aged 6–9, found an association between smaller amygdala GMV and higher aggression scores (Thijssen et al., 2015). As validated by the aggression diathesis model, we speculate this strong correlation may be due to increased aggression leading to an imbalance between top down and bottom up control systems (Siever, 2008). Greater acts of aggression may lead top down systems, which are modulated by brain regions that are highly interconnected with the amygdala, such as the orbitofrontal cortex (OFC) and the anterior cingulate cortex (ACC), to fail to suppress aggressive acts in the presence of anger inducing stimuli (Siever, 2008). Moreover, increased aggression may induce hyperresponsivity of limbic brain regions, such as the amygdala, which provide the “drive” to respond to incoming danger and threat (Siever, 2008).

We developed an ensemble ML model to find brain and behavioral features that were associated with SS exposure on given participants. Our main goal was to see whether certain features contribute accurately to predicting structural changes in brain morphometry from exposure using ML. We achieved the best accuracy by using the feature selection method, RFE. We also examined the effect of oversampling by using SMOTE on all features. For high dimensional data, SMOTE generally performs better by avoiding overfitting (Dessie et al., 2021; Park et al., 2021). However, for data with a very small sample size (*n* = 30), involving augmented (simulation) data provides little benefit if we reduce feature dimensionality because overfitting is no longer a problem and oversampling can lead to underperformance due to a deteriorating sampling (Elreedy and Atiya, 2019). From our best results, we see that with a small data set, with very few positive cases, reducing the dimensionality, rather than introducing synthetic data, leads to higher model performance. Considering achieving greater generalizability is one of our aims, reducing the dimensionality would be one of the critical solutions. Our finding is consistent with prior studies using small sample sizes. Crippa et al. (2015) identified children with autism spectrum disorder (ASD) from a sample size of *N* = 30. Güven et al. (2020) predicted attention-deficit hyperactivity disorder (ADHD) children from a sample size of *N* = 44. Both studies implemented feature selection either manually or algorithmically.

Within the summary SHAP plot, most of the important features that affect our target value came from the brain volumetric data (i.e., the amygdala, hippocampus, and caudate) suggesting the brain’s particular sensitivity to stress exposure. Further, the dependence plots showed distinctly larger amygdala and smaller PHG brain volumes, respectively, increase the likelihood of SS exposure.

The study has various limitations. First, despite our prior research indicating the binary SS index (exposed vs. not exposed *in utero*) to be a significant and critical indicator of impairment, we acknowledge the use of binary, objective indices prevent the analysis of important, fine-grained measures, such as post-traumatic psychosocial reactions to SS. Second, the BASC-2P questionnaire used to assess child behavioral problems was based on parental report, which may lead to subjective bias. However, our study incorporated brain imaging to corroborate and enhance the objectivity and robustness of the findings. Third, information on the handedness of the children was not collected and fourth, the sample size was relatively small with mostly girls. Thus, the results should be interpreted with caution and warrant replication with bigger sample sizes in the future.

There are several strengths in this study. First, our study utilized a quasi-experimental design and rendered the pseudo-randomization of a natural disaster-related stress *in utero*. Currently, most of the studies that have investigated stress during pregnancy relied on the measurement of maternal psychopathology, trauma history or nominal pregnancy stress—which are likely to be confounded by factors, such as genetics, and heterogeneity of level of stress exerted. Second, the current study is part of a larger longitudinal project prospectively tracking and assessing the neurodevelopment of children, preventing the need to rely on retrospective parental reports. This eliminates the problem due to recall bias, which is common in cross-sectional research using information based on retrospective reports in part. Third, our study is the very first to deploy the technique of ML on neurobehavioral and brain imaging data in the research of *in utero* maternal stress. Future studies will aim to expand on our initial findings by tracking changes from childhood into adolescence.

## 5. Conclusion

Exploiting ML, we developed multiple models for classification and chose the RFE algorithm to extract the subset of features that contributed the most optimally to detection of natural disaster exposure *in utero*. The use of SHAP values assisted in model interpretation. SHAP plots provided critical insights into behavioral phenotypes and brain volumetric changes associated with natural disaster exposure *in utero* among offspring, especially within brain regions implicated in emotion regulation (the amygdala, caudate, and PHG). The goal of this study was to leverage advanced computational methodology to understand how a significant stressor connects brain and behavioral development among high-risk populations. Our preliminary results suggest that targeted intervention on behavioral phenotypes, such as aggression, and structural morphological alterations in the emotional centers of the brain can potentially play a key role in buffering the adverse impact of prenatal maternal stress on brain development and improve subsequent developmental outcomes.

## Data availability statement

The raw data supporting the conclusions of this article will be made available by the authors, without undue reservation.

## Ethics statement

The studies involving human participants were reviewed and approved by the City University of New York University Integrated Institutional Review Board (protocol number: 2018-1305-QC). Written informed consent to participate in this study was provided by the participants’ legal guardian/next of kin.

## Author contributions

GD and C-LT led the study and oversaw the integrity of the ML implementation in the study. AS supervised the interpretation of the brain structure findings. YN supervised the interpretation of the behavioral findings. All authors contributed to the drafting, revising, and interpreting the data.
