# Supplementary material for: Superstorm Sandy exposure in utero is associated with neurobehavioral phenotypes and brain structure alterations in childhood: A machine learning approach
Source: Front Neurosci. 2023 Feb 2;17:1113927. doi: 10.3389/fnins.2023.1113927 (PMC9932505; doi:10.3389/fnins.2023.1113927)
Supplement: Supplementary file 1 [file Data_Sheet_1.docx]

Appendix: Performance Study of ML Classification Algorithms

This section provides additional information and support for choosing the machine learning algorithm for this study. Supp 1 presents the performance comparison of three classification algorithms (Random Forest, XGBoost, and AdaBoost) applied to our dataset, based on balanced accuracy and F1 score. To ensure fairness, we set the same model hyperparameters for all the algorithms, and the best outcome was also achieved with the same feature set selected using RFE without data augmentation. In this study, the RF classifier was ultimately chosen due to its superior performance in terms of prediction accuracy.

It is noteworthy that, despite variations in prediction accuracy, all three models yielded the same ranking of feature importance, leading to similar conclusion in term of feature analysis. This further suggests that our ML framework for analysis of features associated with *in-utero* exposure to natural disasters is robust and can be replicated using common ML classification algorithms.

Supp 1: Performance comparison of ML algorithms with 6 features chosen by RFE and no SMOTE data augmentation.

| **#** | **ML Algorithm** | **Balanced Accuracy** | **F1 Score** |
| --- | --- | --- | --- |
| 1 | Random Forest | 0.8667 | 0.7778 |
| 2 | XGBoost | 0.8333 | 0.6667 |
| 3 | AdaBoost | 0.80 | 0.6667 |
